# Grisel's syndrome accompanying a submandibular abscess^☆^

**DOI:** 10.1016/j.bjorl.2016.07.004

**Published:** 2016-08-23

**Authors:** Onur Ismi, Hakan Ozalp, Vural Hamzaoglu, Helen Bucioglu, Yusuf Vayısoglu, Kemal Gorur

**Affiliations:** a University of Mersin, Faculty of Medicine, Department of Otorhinolaryngology, Mersin, Turkey; b University of Mersin, Faculty of Medicine, Department of Neurosurgery, Mersin, Turkey

## Introduction

Grisel's syndrome (GS) is the non-traumatic atlantoaxial joint subluxation firstly described by Pierre Grisel in 1951 in two patients with pharyngitis.^1^ Traumatic subluxations or underlying bone diseases are not considered as GS. It is mostly seen in the pediatric age group. Upper respiratory tract infections and common otolaryngologic surgical procedures such as adenotonsillectomy are predisposing factors.^2^ Delayed diagnosis can cause neurological sequela and may need neurosurgical interventions such as posterior arthrodesis.^2^

In case of GS seen after upper respiratory tract infections, presenting symptoms include fever, torticollis, and pain during head maneuvers. GS seen after adenotonsillectomy needs meticulous suspicion, because torticollis and pain in neck movements can be attributed to postoperative pain which can lead to delayed diagnosis.^3^ Since the corpus of C1 and C2 are close contact with prevertebral and retropharyngeal areas, abscess or cellulitis of these areas can also cause GS.^4^, ^5^, ^6^

In this case report, we presented a pediatric patient with GS accompanying a submandibular deep neck abscess. The importance of early diagnosis with great suspicion is discussed in this case report under the light of current literature. To the best of our knowledge, we presented the first case of Grisel's syndrome seen together with a submandibular abscess.

## Case report

An eight years old patient was referred to our tertiary center otorhinolaryngology clinic with the complaint of trismus, fever, neck edema and limited neck movements. In his past medical history, he was hospitalized in another hospital for upper respiratory tract infection with tonsillopharyngitis and treated with intravenous antibiotics. After two days of hospitalization he had got worse with left submandibular swelling, trismus and limited neck movements.

**Figure 1:**
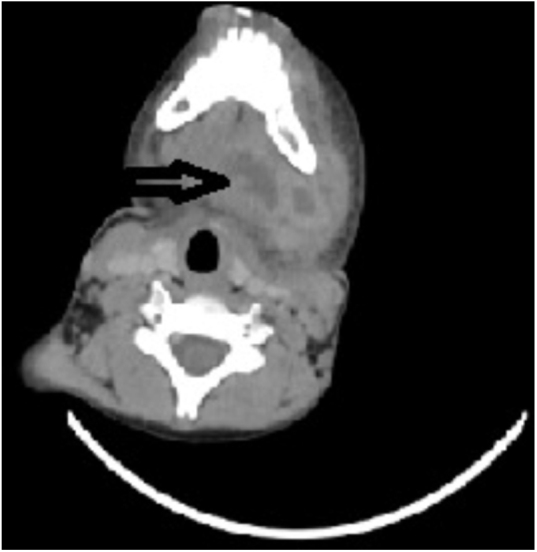
Computerized tomography of the patient was presented (black arrow shows the submandibular abscess).

After he was then referred to our clinic, he had approximately 5 cm swelling in the left submandibular region, trismus with 38.3 °C fever and 15 × 10^3^ μL white blood count. Neck CT revealed a 4 × 2 cm left submandibular and submental abscess without involvement of prevertebral or retropharyngeal area (Fig. 1).

**Figure 2:**
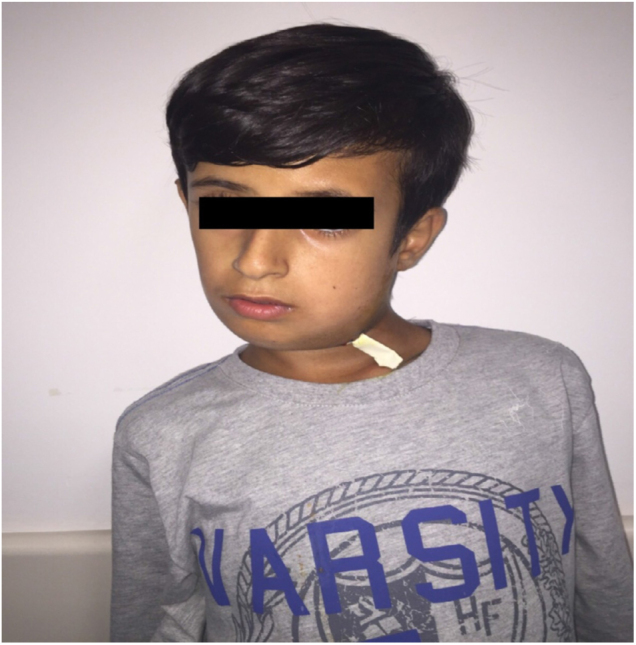
Clinical appearance of the patient with the head deviated to left and chin tilted to right (cock-robin position) has been demonstrated. The drain that was placed in the surgical field during abscess drainage was also seen.

**Figure 3:**
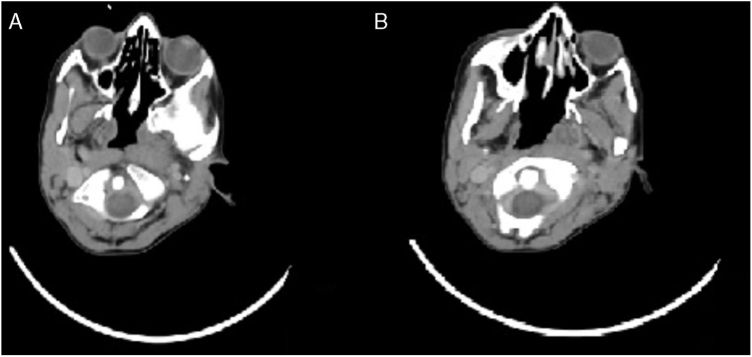
(A) The body of C1 was presented on the computerized tomography. (B) Computerized tomography demonstrates the atlantoaxial rotary subluxation.

Submandibular abscess was drained under general anesthesia. Two days postoperatively, his neck swelling and trismus diminished, his head was still tilted to left side with limited neck movements and his chin was deviated to right side (Fig. 2). GS was suspected and when the CT was overlooked again, rotationary subluxation of C1 and C2 was seen which was omitted previously (Fig. 3). MR imaging and neurosurgery department consultation was performed. Transverse and lateral ligaments were intact in MR, these findings were well consistent with a Type I Fielding classification^7^ GS.

Philadelphia collar was suggested for three weeks by neurosurgery department. After two weeks course of intravenous sulbactam-ampicilline 1 g two times a day treatment, the patient was discharged. On the first month visit, he was well without neck immobility.

## Discussion

Grisel syndrome is a rare complication of upper airway infections or head and neck surgeries which the ENT surgeons must be aware of and deal with.^8^ The most common causes are infections including pharyngitis, adenoiditis, tonsillitis, otitis media or ENT operations such as adenotonsillectomy, mastoidectomy, choanal atresia repair and excision of neck tumors.^2^ The predominant cause differs in the literature. In the review of Karkos et al.^3^ vast majority of the cases were reported to be infection related (48%), in the series of Deichmueller et al.^8^ most of the cases (67%) were related to ENT operations.

Gender or side predominance has not been reported yet.^9^ GS is a disease of childhood, because upper airway infections are more common compared to adults. Children also have larger head size in relation to the trunk, weaker cervical muscles, looser ligaments, shallower and more horizontally placed C1–C2 joint compared to adults which make them prone to atlantoaxial subluxation.^2^

The exact underlying pathogenesis of GS is not known. Whatever the underlying cause is, this insult results in hyperemia and pathological relaxation of the ligaments of the atlantoaxial joint.^2^ Hyperemia secondary to inflammation leads to decalcifications of the anterior sac of the atlas and laxity of the anterior transverse ligament.^10^ The inflammation of atlantoaxial joint with a nearby infection such as prevertebral area abscess with tuberculosis,^11^ retropharyngeal cellulitis^5^ or abscess^6^ can cause laxity in the joint and subsequent subluxation. But this phenomenon does not explain the GS cases with minor distant infections such as pharyngitis or otitis media.

Parke et al.^12^ demonstrated the novel pharyngovertebral veins which cross through posterior pharyngeal wall and nasopharynx to periodontial plexus and subsequently atlanto-occipital membranes on both sides. These veins provide a hematogenous septic effusion of a pharyngeal infection to reach the atlantoaxial joint causing subluxation.^9^ Battiata et al.^9^ proposed a two-hit hypothesis for explaining pathogenesis of GS. In their hypothesis, the first hit is the preexisting cervical ligamentous laxity seen in pediatric age group at baseline. Second hit is the inflammatory process causing cervical muscle spasm and subsequent subluxation by transmission of inflammatory mediators to cervical muscles and atlantoaxial joint with pharyngovertebral plexus.

For our patient previous history of tonsillopharyngeal infection was the prominent cause of the submandibular abscess. The major cause of the GS was also thought to be tonsillopharyngitis rather than the submandibular abscess itself. Beside primary metastatic inflammatory effusion, muscular spasm secondary to inflammation can also cause subluxation of the joint.^6^

Clinical sign and symptoms of GS include neck stiffness and pain on attempted position and dysphagia. Patients are mostly in fever. The head is tilted to the side of the subluxation, whereas chin looks at the opposite side called as the cock-robin position.^13^ Spasm of the Sternocleidomastoid muscle (SCM) and torticollis is frequent. Sudeck's sign is the palpation of spinous process of C2 displaced to the same side toward the head^9^ and it is mostly seen in GS cases.

Our patient was also in the cock-robin position. Neck stiffness and pain are non-specific symptoms, if GS occurs after adenotonsillectomy, these symptoms can be attributed to the postoperative pain which can cause delayed diagnosis.^3^ In case of a submandibular abscess, as in our case, neck swelling and stiffness can hide an underlying GS. Edema of the neck fascias can obscure the SCM muscle spasm and torticollis.

For the management of our case, we were unaware of the GS at first admission, symptoms of neck swelling, trismus and pain were attributed to his primary diagnosis of deep neck abscess, and we drained the neck abscess under general anesthesia without using any collar. Although any complication did not occur in our case; surgical manipulation or neck extension during tracheal intubation could make a hazardous complication such as spinal cord compression and neurologic deficit.

Fielding and colleagues^7^ proposed a grading system for choosing treatment options in GS. Most authors advise bed rest, muscle relaxants, non-steriodal anti-inflammatory drugs and neck immobilization with soft collars for Type I and II cases.^13^ More aggressive options such as halo immobilization, cervical traction, surgery including arthrodesis and C1–C2 cervical fusion are in option for Type III and IV cases.^11^

Early diagnosis of GS is important, because delayed diagnosis may lead to irreducible subluxation due to atlantoaxial rotatory fibrosis which is amenable to surgery rather than conservative treatment options.^2^ Our case was a Type I GS, who was successfully treated with a neck immobilization with soft collar. For GS cases neurological complications can occur in up to 15% of cases, with extreme consequences including quadriplegia and sudden death. Type III and IV have a higher risk of neurological sequela.^13^

## Conclusion

As a conclusion, this first report of GS accompanying a submandibular abscess must be kept in mind for patients admitting with complaint of trismus, neck pain and stiffness. Although they address the submandibular abscess as a causative factor, these non-specific symptoms can hide an underlying Grisel's syndrome. Preoperative neck computerized tomography scans must be evaluated carefully not to omit this rare disease.

## Conflicts of interest

The authors declare no conflicts of interest.
